# A Systematic Review and Meta-Analysis of Catheter Ablation Versus Anti-arrhythmic Drugs for Treatment of Ventricular Arrhythmia

**DOI:** 10.7759/cureus.67649

**Published:** 2024-08-24

**Authors:** Atif Rashid, Mohammad Faisal Khan, Javed Rashid

**Affiliations:** 1 Cardiothoracic Surgery Department, Fortis Hospital, Kolkata, IND; 2 Emergency Department, New Bombay Hospital, Mumbai, IND

**Keywords:** anti-arrhythmic drugs, implantable cardioverter defibrillator, catheter ablation, ventricular arrhythmia, meta-analysis, systematic review

## Abstract

Catheter ablation (CA) and anti-arrhythmic drugs (AADs) minimize implanted cardioverter-defibrillator (ICD) shocks in individuals with ischemic cardiomyopathy and an ICD, while the best strategy is still unknown. CA has been proposed as a potentially effective means of reducing the occurrence of ICD events in a number of studies; however, there were insufficient relevant dates from randomized controlled trials. A meta-analysis and systematic review of randomized controlled trials were carried out to evaluate the efficacy of CA for the prevention of VA in patients with ischemic heart disease. Cardiovascular mortality, an unscheduled hospitalization due to increasing heart failure, appropriate ICD shock, or serious treatment-related consequences comprised the composite primary outcome. AADs were examined in six trials (n = 1564; follow-up = 15 ± 8 months), while CA was evaluated in four trials (n = 682; follow-up = 12 ± 6 months). Both CA (odds ratio (OR) 0.65, 95% confidence interval (CI) 0.47-0.82, p = 0.001) and AADs (OR 0.76, 95% CI 0.32-0.84, p = 0.034) significantly reduced the number of suitable ICD interventions, with no discernible difference between the two treatment approaches. AADs were observed to reduce incorrect ICD interventions (OR 0.38, p = 0.001), but CA did not. During follow-up, there was no correlation seen between reduced mortality and either CA or AAD. When compared to AAD, CA decreased the composite endpoint of cardiovascular death, adequate ICD shock, heart failure-related hospitalization, or severe treatment-related consequences in ICD patients with ischemic cardiomyopathy and symptomatic VT.

## Introduction and background

Ventricular arrhythmias (VA) are irregular heartbeats that come from the heart's lower chambers. The heart may beat excessively fast as a result of these arrhythmias, which may stop oxygenated blood from reaching the body and brain. It may result in cardiac arrest. Ventricular fibrillation (VF), ventricular tachycardia (VT), and preventricular contractions (PVC) are the three major forms of VA.

Although adequate shocks from implanted cardioverter-defibrillators (ICDs) prevent sudden cardiac death, they are uncomfortable, worsen the quality of life (QoL), shorten the device's lifespan, and may potentially increase mortality [1-6]. Randomized trials have demonstrated that CA and AADs both lower appropriate ICD shocks in patients with ischemic heart disease [7-9]. VT is effectively cured by the ICDs, but recurrent arrhythmias and ICD shocks can lower QoL [10], increase heart failure, hospitalization, and the risk of death, and necessitate suppressive therapy, most frequently the use of AADs, in many cases. Patients and clinicians must decide between CA and increasing medication therapy if VT recurs despite AAD therapy [11].

In individuals experiencing VT recurrences while receiving amiodarone, ablation appears to be a more effective treatment than increasing AADs; however, no randomized study has examined the effectiveness and safety of both interventions in patients who are new to AADs [12]. AADs most likely have fewer acute problems, but they can nevertheless have serious side effects and raise mortality in certain patient subgroups [12,13].

Randomized trials have estimated that CA for VT lowers the recurrence rate [7,8], and multicenter observational studies have linked a higher survival rate to the absence of VT following CA [14]. Patients with an ICD and ischemic cardiomyopathy who experienced VT despite first-line AAD medication were compared CA with escalated AAD therapy in the VT ablation versus escalated AAD in the ischemic heart disease (VANISH) trial.

ICD shocks have been linked to higher death rates [15,16] and lower QoL [17-20], according to further investigations. In the VANISH study, CA was found to be superior to escalation of AAD for patients with ischemic cardiomyopathy and VT despite first-line AAD therapy. This resulted in a 28% relative risk reduction in a composite endpoint of mortality, ICD shock, or VT storm [12]. In that study, patients who developed VT while receiving amiodarone (n = 169) or non-amiodarone anti-arrhythmic drugs (AADs) (sotalol, n = 89; procainamide, n = 1) were enrolled; patients were randomized based on whether they had VT despite receiving amiodarone (amino-refractory) or not (sotalol-refractory); patients who were sotalol-refractory were randomly assigned to receive either amiodarone or catheter ablation (CA), while those who received amiodarone-refractory were assigned to receive either escalated medication dosing (addition of mexiletine or amiodarone reloading and higher dosage) or CA [21].

The substrate ablation versus AAD therapy for symptomatic VT (SURVIVE-VT) trial was designed to evaluate substrate-based CA versus AADs as the initial treatment for patients with ICD shocks or symptomatic VT and ischemic cardiomyopathy [22]. The goal was to determine a composite efficacy and safety outcome. In patients with SHD-related VT, the catheter ablation versus AADs for ventricular tachycardia (CAAD-VT) trial will assist in determining if CA is superior to antiarrhythmic medication therapy alone [23].

Therefore, the current meta-analysis of randomized controlled studies was intended to evaluate the safety and efficacy of CA versus AAD and to further confirm whether or not it could prevent VA storm, lower ICD therapy, and mortality in patients with ischemic heart disease.

## Review

Methods

Throughout the entire process, we adhered to the Preferred Reporting Items for Systematic Reviews and Meta-Analyses (PRISMA) criteria at every stage of design and execution [24].

Search strategy

We performed a language-free literature search on Embase, PubMed, Google Scholar, and Cochrane Library. We were restricted to human studies and randomized controlled trials, and the keywords we used were "ventricular arrhythmia," "ventricular tachycardia," "ischemic heart disease" or "implantable cardioverter defibrillator," and "catheter ablation" or "anti-arrhythmic drugs." Furthermore, we gathered the study scripts from those that weren't initially recovered and analyzed the pertinent reviews to search for any possible missing studies.

Selection criteria

Studies were specifically considered for inclusion if they satisfied the subsequent inclusion criteria: (1) AADs versus CA in a randomized controlled trial; (2) investigating ICD patients; (3) a comparison of standard medical therapy versus AAD or control medical therapy versus CA; (4) at least three of the endpoints listed below were reported by the study: ICD therapy, VA storm, all-cause mortality, hospitalization related to cardiovascular disease, and consequences. All randomized controlled studies met the National Library of Medicine's guidelines for eligible trials. There was no evaluation of the reproducibility and inter-observer agreement of the study selection and assessment procedures. Exclusion criteria included studies that enrolled individuals with non-ischemic heart disease, animal experiments, and non-randomized controlled trials.

Extraction of data and assessment of results

The main outcome under investigation was whether or not AADs or CA prevented recurrent VA, which may include AADs in trials assessing CA. Data on inclusion and exclusion criteria, totally randomized patients, total ICD patients, number of follow-up VA episodes (measured as the quantity of suitable ICD interventions, including anti-tachycardia pacing (ATP)) or ICD shocks, length of study follow-up, and complications. The total mortality during further investigation and the quantity of ineffective ICD therapies were taken out of the studies and incorporated into the meta-analysis. ICD therapy, ICD shock, and VA storms were the main outcomes of interest. Hospitalization linked to cardiovascular disease, complications, and all-cause mortality were additional outcomes.

Quality assessment

Six dominants were included in RCTs using the Cochrane risk of bias (ROB) tool: allocation concealment, random sequence generation, participant and professional blinding, selective reporting, insufficient outcome data, and other biases. There are three categories for the evaluation results: unclear, low risk, and high risk [25]. It was too challenging to carry out the blind technique since the included RCTs compared the effect of CA on the prevention of VT in patients with IHD. This could have led to a significant risk of statistical bias in the research investigations. Assignment concealment and masking were not feasible in the initial studies since one of the studied therapies, namely CA, constituted an intrusive procedure [26]. As a result, these items were not included in the quality evaluation of studies assessing CA in the current study. However, almost all of the studies were at unclear or low risk in terms of detection, selection, reporting, and attrition bias. 

Statistical analysis

STATA MP15 (Stata Corp., LLC, College Station, United States) was used to conduct the statistical analysis. A 95% confidence interval (CI) and odds ratios (OR) for binary variables were aggregated and presented. A random-effect model [27] with inverse-variance weighting was used to compute the summary effect estimate, and a difference was determined to be statistically significant [25]. I-square statistics were used to assess the statistical heterogeneity between the studies and were deemed to indicate no significant heterogeneity, whereas they were considered to indicate considerable heterogeneity. Using Kaplan-Meier product-limit estimations, survival rates were summed up for the groups receiving escalated AAD therapy and CA based on their initial AAD therapy.

Results

Extraction of Articles

**Figure 1:**
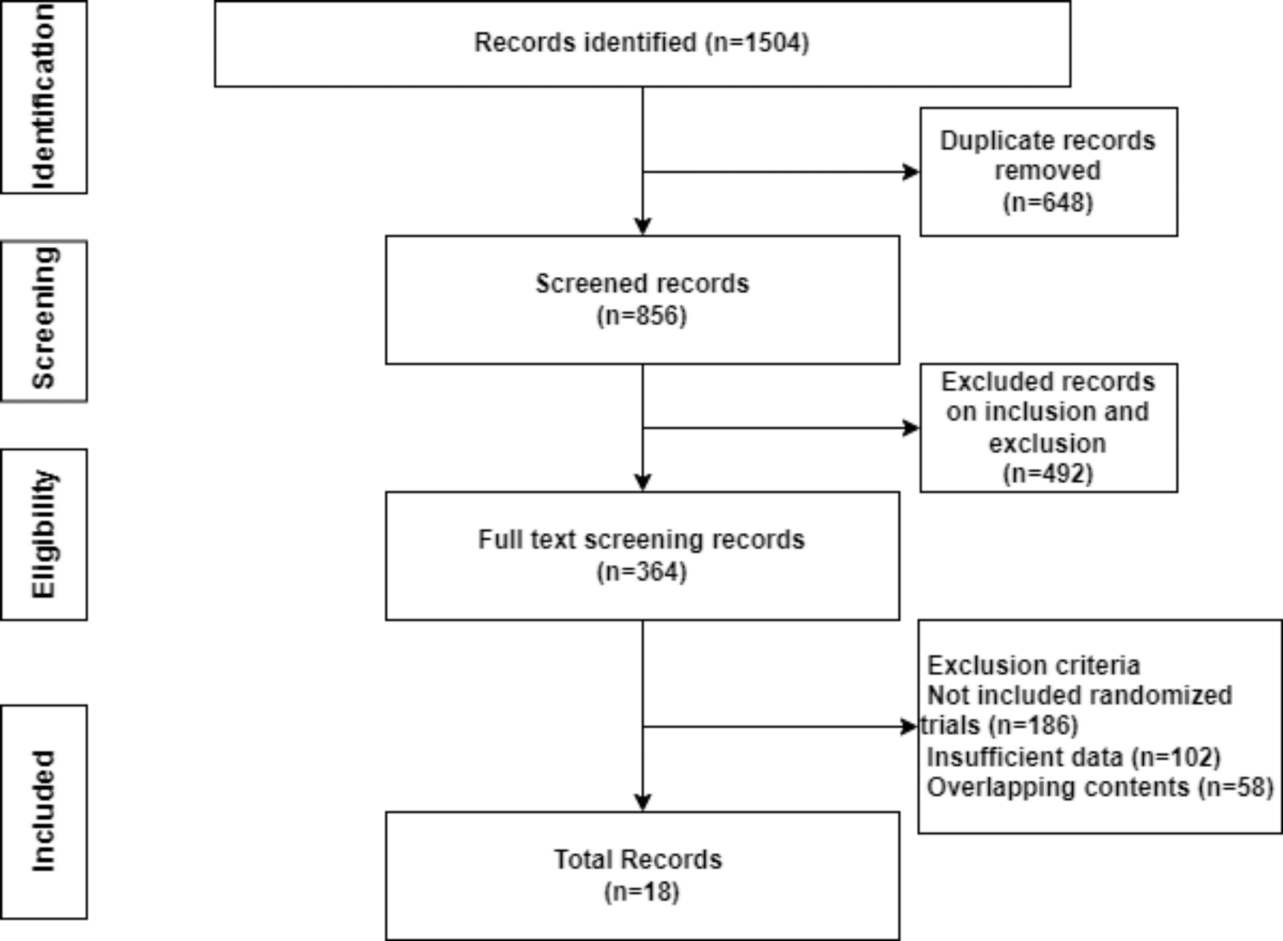
Selecting studies for the meta-analysis using PRISMA PRISMA: Preferred Reporting Items for Systematic Reviews and Meta-Analyses

A total of 1504 articles were retrieved for the search. About 856 prospective studies were taken into consideration for additional screening after duplicate citations were removed. The studies that were chosen for additional examination were all written in English. After the complete text was evaluated, 492 studies were excluded. Non-randomized trials resulted in the exclusion of an additional 186 studies. Out of the randomized trials, 58 studies had overlapping subject matter, and 102 studies were eliminated because they exclusively included individuals without a CA, AAD, VA, or ICD. In the end, we were able to locate 18 prospective randomized studies that satisfied the requirements for inclusion and were added to the meta-analysis. Figure 1 depicts a flow diagram of the selection procedure. Supplemental Table 1 presents the baseline characteristics of the trials that were part of the meta-analysis as well as the study quality evaluation. Every study met the inclusion criteria and used a randomized controlled methodology.

**Table 1:** Results of CA and AAD based on VA AAD: anti-arrhythmic drug; ICD: implantable cardioverter defibrillator; MI: myocardial infarction; SURVIVE-VT: substrate ablation versus AAD therapy for symptomatic VT; VT: ventricular tachycardia; CA: catheter ablation

References	Quality assessment	Ablation	AAD	Hazard ratio	p-value	Primary research	Outcome evaluation
Arenal et al. [22]	SURVIVE-VT	68.5 (57.8-81.1)	46.2 (35.1-60.8)	0.52	0.019	Anti-arrhythmic drug (AAD) (73 patients) or catheter ablation (71 patients) was the randomization strategy used for 144 patients (96% male; median age, 70 years).	A combination of severe treatment-related problems, unplanned admission due to deteriorating heart failure, appropriate implantable cardioverter defibrillator (ICD) shock, and cardiovascular death.
Sapp et al. [12]	Kaplan-Meier	78 (59.1)	52 (61.2)	0.72	0.04	Out of the 259 enrolled patients, 132 were placed in the ablation group and the remaining 127 in the escalated therapy group.	Catheter ablation had a significantly lower rate of death, ventricular tachycardia (VT) storm, or appropriate ICD shock than escalation in ischemic cardiomyopathy and ICD patients with VT despite antiarrhythmic treatment.
Parkash et al. [21]	Kaplan-Meier	23 (27.1)	35 (41.7)	0.53	0.020	VANISH was a blinded clinical trial comparing increased antiarrhythmic medication therapy in 259 patients with past myocardial infarction (MI), VT, and an ICD.	Individuals with amiodarone-refractory VT benefited more from catheter ablation than those switched to amiodarone, and they also had a higher rate of mortality and ventricular arrhythmia.
Avila et al. [28]	Markov Chain Monte Carlo	67.6	48.4	19.2	0.75	Of the 144 randomized patients in the SURVIVE-VT trial, 71 had catheter ablation and 73 received AAD.	For CA, the absolute variations were 15.2%, 21.2%, and 20.2%, respectively, with a high probability (>93%) of lowering unnecessary hospitalizations for ventricular arrhythmias, incessant/slow undiagnosed VT/electric storm, and total cardiovascular admissions > 25%.
Samuel et al. [29]	Anderson-Gill recurrent event model	85 (64.4)	84 (66.1)	0.80	0.05	Patients with prior VT and MI despite initial AAD therapy were randomized to either catheter ablation or increased AAD medication in the VANISH study. 129 patients to increased AAD therapy and 132 patients were randomly assigned to ablation out of the 259 enrolled patients (7.0% women (69.8 years)).	When compared to escalated AAD therapy, CA decreased the appropriate shock pressure and shock-treated VT event burden in patients with a prior MI and AAD-refractory VT.
Coyle et al. [30]	VANISH	1.63 (1.41-1.85)	1.49 (1.26-1.75)	0.14	-	The study compared the cost-effectiveness of CA (n = 132) with escalating AAD treatment (n = 127) using randomized controlled trial methodology.	Comparing ablation to pharmacological therapy escalation, ablation is more affordable.
Della Bella et al. [31]	Kaplan-Meier	0.73	0.86	0.83	0.80	This randomized trial trial included 124 consecutive patients with recurrent, drug-refractory, hemodynamically tolerated ventricular tachycardia.	In 91 cases (73%), the ablation was successful in ending the sustained VT; in 21 cases (17%), a partial outcome was achieved, and in 12 cases (10%), there was a failure. In 86% of the patients, low doses of amiodarone and/or beta-blockers were sustained.

Comparative Effectiveness of CA Versus AADs to Prevent Recurrent VA

For patients with inferior MI, the composite primary outcome of all-cause mortality, proper VT storm, and ICD shock (95% CI-0.51-0.20, hazard ratio (HR) 0.80) did not show a statistically significant difference between treatment arms. However, in patients with non-inferior Mis, ablation dramatically reduced the incidence of the primary outcome (95% CI 0.27-0.86, HR 0.48). Anterior MI patients (n = 83; 95% CI 0.23-1.09, HR 0.50) in a sensitivity study showed a trend toward a decline in the primary outcome following ablation [32].

Additionally, due to extended VT inducibility, a greater number of patients in the off-amiodarone group than in the on-amiodarone group required epicardial ablation (13/50 (26%) versus 5/84 (6%), respectively; p < 0.001). In the on-amiodarone group, the recurrence of any VA off AAD was 44% (37/84) but in the off-amiodarone group, it was 22% (11/50) (p = 0.013) [33]. The follow-up period averaged 23.9 ± 11.6 months.

For patients with symptomatic VT and ischemic cardiomyopathy or ICD shocks, the SURVIVE-VT trial compared substrate-based CA with AAD as first-line therapy [28]. The incidence of recurrent VT was not significantly different between the two methods in an indirect comparison of CA and AADs. The OR for CA versus AADs was 0.65 with a 95% CI of 0.47 to 0.82, and a p-value of 0.001, suggesting that CA may be associated with a lower likelihood of recurrent VT compared to AADs. In comparison to AADs, CA was linked to a non-significant incremental benefit when studies with amiodarone were excluded, OR was 0.76 with a 95% CI of 0.32 to 0.84 and a p-value of 0.034. This indicates that, excluding studies where amiodarone was used, CA might provide a benefit in reducing recurrent VT, though the result is not statistically significant. Similar risks of all-cause death were linked to the two treatment approaches, OR was 0.43 with a 95% CI of 0.36 to 1.28, and a p-value of 0.109, indicating no significant difference in mortality between the two treatment approaches.

**Figure 2:**
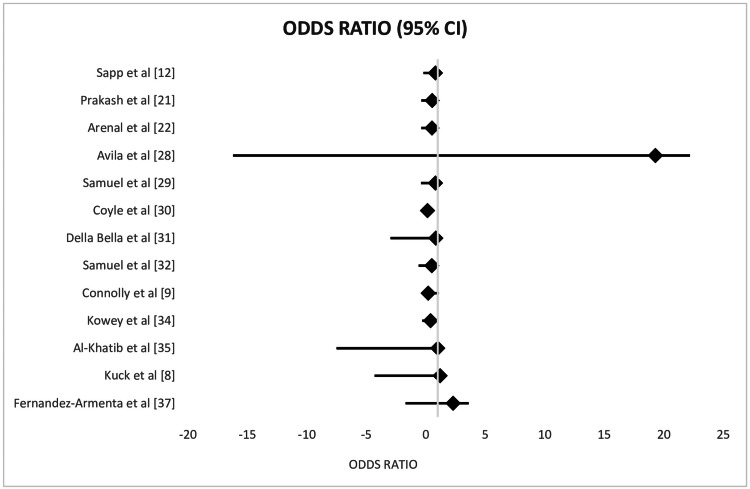
Forest plot comparing CA and AAD, exhibiting individual and pooled OR with 95% CI for VA therapy CA: catheter ablation; AAD: anti-arrhythmic drug; CI: confidence interval; VA: ventricular arrhythmias

The forest plot in Figure 2 compares AADs with CA for the treatment of VA, displaying individual and pooled OR with 95% CI [8,9,12,21,22,28-32,34-36].

Discussion

The purpose of this study was to evaluate and compare the effectiveness of CA and AADs in reducing the risk of recurrent VA in patients with ICD. Its foundation is the statistical pooling of more than 1500 participants from 18 randomized controlled studies. Both AADs and CA significantly decrease the incidence of recurrent VA, and appropriate ICD interventions are observed as compared to control medical therapy. The two treatment approaches did not differ significantly from one another. Studies employing amiodarone were the main source of evidence supporting the usefulness of AADs in avoiding VA episodes. Moreover, all-cause mortality was not significantly affected by AADs or CA, despite the fact that they substantially reduced the number of VA events.

Moreover, 95% CIs and effect estimates are frequently utilized to provide details regarding the size of the effect. However, because they do not indicate the likelihood that the genuine effect falls within these areas, the CIs are frequently interpreted incorrectly. According to the frequentist paradigm, a study's computed CI either contains the true value or does not. About 95% of the samples would contain the true value if we were to generate numerous CIs from different samples [37]. This is the correct interpretation. The forest plot in Figure 2 compares AADs with CA for the treatment of VA, displaying individual and pooled OR with 95% CI 

CA had a high chance (>93%) of lowering unnecessary hospitalizations for VA, incessant/slow undiagnosed VT/electric storm, and total cardiovascular admissions >25% [31]. Out of the 1990 patients, 740 got radiofrequency ablation that was greater than 1.4 ± 0.9 (range: 1-10). VT storm (38% versus 33%) or ICD shocks (70% versus 63%), and had received treatment with ≥2 antiarrhythmic medications (22% versus 14%) or amiodarone (55% versus 48%) [38].

Compared to AAD, CA as a first-line therapy had a high likelihood of lowering various clinical outcomes in individuals with VT and ischemic cardiomyopathy. When compared to escalated AAD therapy, CA decreased the appropriate shock load and shock-treated VT event burden in patients with AAD-prior MI and refractory VT. Patients receiving ablation also had reduced VT burden, ATP-treated VT event load, and adequate ATP burden; however, this benefit was restricted to those with amiodarone-refractory VT. The current substudy's findings quantify the impact of CA for VT suppression in individuals with AAD-refractory VT and a history of MI [32]. As previously reported [7], the SURVIVE-VT trial provides additional evidence that AAD and CA are both very successful in preventing ICD shocks and therapies. By preventing serious AAD-related problems, substrate-based CA dramatically lowered the primary endpoint [39,40]. Based on these findings, individuals with ischemic cardiomyopathy and VA should seriously consider CA as a first-line treatment option prior to AAD.

QoL outcomes are critical in evaluating the overall clinical effectiveness and patient preference for CA versus AAD therapies, as they influence long-term adherence and patient satisfaction. Long-term rates of cardiac mortality and sudden death are extremely low for patients with recurrent tolerable VT treated with radiofrequency CA. An ICD implant and/or repeat ablation are necessary if persistent VT inducibility remains following CA [41]. Ultimately, the meta-analysis verified that AADs had a function in preventing unwarranted ICD treatments; this is probably due to a decrease in supraventricular tachyarrhythmia frequency and an improvement in rate control during arrhythmia episodes.

Limitations

The limitations of this meta-analysis are numerous. VA recurrence, which we defined as persistent VT resulting in appropriate ICD intervention, was the main outcome of our study. It was not consistently possible to extract separate data on the number of shocks versus anti-tachycardia pacing treatments from the available trials. It was appropriate to use anti-tachycardia pacing in addition to ICD shock. Furthermore, given that current ICD programming tactics have been shown to reduce the risk of both suitable and improper ICD interventions as well as death, the reported findings might not apply to contemporary cohorts of ICD patients. Unlike frequentist approaches, there are no predetermined levels of statistical significance; “for a moderate/great reduction in an endpoint and for a clinically relevant reduction” within this specific clinical context are instances of what we considered clinically noteworthy, and the criteria utilized in the clinical hypothesis were subjective [28]. Nonetheless, there's still a chance that the three-year follow-up won't reveal how the two treatment approaches differ in terms of adverse outcomes. Specifically, it might overlook the disease's advancement within the ablation group and might overlook the unfavorable effects of the medication, which usually manifest themselves somewhat later in amiodarone-treated patients. The detection of recurrent VA episodes was probably improved by including studies with more conservative ICD programming, which made it easier to assess the effectiveness of antiarrhythmic medications or CA in preventing VA. The identified limitations impact the interpretation and generalizability of findings on CA and AAD therapies, underscoring the need for detailed, standardized, and long-term research. The lack of significant impact on cardiovascular and all-cause mortality suggests that while these therapies effectively manage arrhythmias, comprehensive patient care must address broader health factors. It is crucial to note, though, that our review's main objective was to examine how various therapy approaches affected VA recurrence compared to ICD shocks. Moreover, variable CA success rates resulting from various ablation techniques and intervals between follow-up visits may also influence the incidence of VA recurrence. Last, the included studies did not compare the two groups' quality assessment of life, which was frequently a highly significant predictor of clinical success, making it impossible for the current meta-analysis to further summarize and interpret the data.

## Conclusions

This comprehensive review presents a meta-analysis of data from 18 randomized-controlled trials assessing CA versus AADs for the prevention of recurrent VA in ICD patients. It demonstrates that when compared to control medical therapy, both CA and AADs (amiodarone) reduce the risk of recurrent VA, with no discernible differences between the two therapeutic approaches. Reductions in improper ICD therapies are also linked to AADs. In conclusion, combined ICD and CA use for VA in patients with ISH disease could significantly lower the incidence of ICD therapy, VT storm, ICD shock, and cardiovascular-related hospitalization; this finding also suggested that CA may eventually prove to be a useful clinical treatment option. However, it was not able to significantly lower the risk of cardiovascular mortality, all-cause mortality, or complications. When compared to AAD, CA decreased the composite endpoint of heart failure-related hospitalization, cardiovascular death, adequate ICD shock, or significant side effects of medication in patients with ICD who have ischemic cardiomyopathy and symptomatic VT.
